# Mononeuritis multiplex as the first presentation of refractory sarcoidosis responsive to etanercept

**DOI:** 10.1186/s12883-014-0237-5

**Published:** 2014-12-11

**Authors:** Inês Brás Marques, Gavin Giovannoni, Monica Marta

**Affiliations:** Queen Mary University London, Blizard Institute, 4 Newark Street, London, E1 1AT UK

## Abstract

**Background:**

Several disorders may present with mononeuritis multiplex and the etiological diagnosis can be challenging.

**Case presentation:**

We report a 42 year-old female who presented with severe lower limb neuropathic pain, asymmetric weakness and sensory impairment and was diagnosed with mononeuritis multiplex. Biopsy showed a granulomatous vasculitic process with eosinophils, scarce granulomata and axonal neuropathy and granulomatosis with poliangiitis was assumed. Steroids, cyclophosphamide, alemtuzumab, azathioprine, mycophenolate mofetil and rituximab were used, all with transient and insufficient response. Skin biopsy performed in a further exacerbation allowed sarcoidosis diagnosis. Infliximab and, later, adalimumab induced good clinical and laboratorial response, but neutralizing antibodies developed to both drugs, so etanercept was tried with good clinical response.

**Conclusions:**

To the best of our knowledge, this is the first report of sarcoidosis successfully treated with etanercept. This drug may be considered in refractory sarcoidosis after other TNF-α inhibitors failure, having the advantage of not being associated with neutralizing antibodies development.

## Background

Mononeuritis multiplex (MM) can be a manifestation of several disorders including infectious, inflammatory, neoplastic, toxic, metabolic and hereditary conditions, and the etiological diagnosis may be challenging. Rarely, it can be a presentation of sarcoidosis, an inflammatory multisystem granulomatous disease that can involve any part of the nervous system.

Peripheral neuropathy is an uncommon manifestation of sarcoidosis patients and presents more frequently with symmetric axonal sensorimotor polyneuropathy, however other manifestations are described, including MM, multifocal motor neuropathy, Guillain-Barré syndrome, polyradiculopathy, lumbosacral plexopathy, small fibre neuropathy and multiple painful sensory mononeuropathies [1-7]. Reports of initial presentation with MM are rare [3,8,9].

Sarcoid neuropathy treatment can also be challenging and, in patients refractory to steroids and imunossupressants, tumor necrosis factor alpha (TNF-α) inhibitors are invaluable [10,11]. According to literature, infliximab and adalimumab, which bind both soluble and membrane bound TNF-α, seem to be more effective in sarcoidosis than etanercept, which binds only to soluble TNF-α with incomplete inhibition of TNF-α bioactivity [12,13].

We report a 42-year old female presenting with MM who was eventually diagnosed with sarcoidosis. Tumor necrosis factor alpha (TNF-α) inhibitors were used after steroid and immunosuppressants failure. As neutralizing antibodies (NAbs) against anti-TNF-α antibodies developed, etanercept was tried with good clinical response.

This case illustrates how sarcoidosis diagnosis and treatment can represent a challenge and is, to the best of our knowledge, the first report of sarcoidosis successfully treated with etanercept.

## Case presentation

A 42 year-old Afro-Caribbean female presented with severe pain in the lower limbs associated with distal weakness, with progressive worsening during the previous week.

Her past medical history was remarkable for longstanding pigmented skin nodules in limbs and torso, bilateral breast implants ten years prior and giving birth to her first child at 26 weeks three months before.

Neurological examination revealed tetraparesis (distal upper limbs: grade 4+/5; proximal lower limbs: right = grade 4/5, left = 3/5; distal lower limbs: right = grade 3/5, left = 2/5), absent ankle reflexes, indifferent plantar reflexes, reduced positional sense in left ankle and up to right knee and reduced vibration and superficial pain sense up to both knees. Physical examination identified multiple hyperpigmented small nodules over the limbs and trunk.

### Investigations

Blood workup revealed normocytic anemia, thrombocytosis and increased erythrocyte sedimentation rate (ESR) (110 mm/h), C-reactive protein (CRP) (33 mg/L) and angiotensin converting enzyme (ACE) (74 units/L). Biochemistry including ionogram, calcium, renal and liver function was unremarkable. Syphilis, hepatitis A, B and C and Human Immunodeficiency Virus serologies were negative. Autoimmune studies showed positive antinuclear antibodies (titre > 1/640, speckled pattern) and anti-neutrophil cytoplasmic antibodies (ANCA) with an atypical cytoplasmic-ANCA (c-ANCA) pattern, however without myeloperoxidaseA (MPO) or proteinase 3 (PR3) specificity. Antinuclear antibodies, antibodies against double stranded DNA, antibodies against extractable nuclear antigens, anti-cardiolipin antibodies and lupus anticoagulant test were negative.

Cerebrospinal fluid showed hyperproteinorraquia (500 mg/L), 5 leukocytes, normal glucose, increased ACE (1.47 units/ml). Direct microscopy, acid-fast bacilli smear, cultures, including mycobacterial culture, and Herpesvirus family and Adenovirus DNA were negative.

Brain and spinal cord magnetic resonance imaging revealed slight pachymeningeal thickening and enhancement over the vertex. Chest, abdomen and pelvis computerized tomography recognized left breast implant intracapsular rupture and mildly enlarged bilateral axillary lymph nodes only.

Nerve conduction studies identified patchy asymmetrical involvement of sensory nerves in upper and lower limbs with axonal involvement (both sural and superficial peroneal nerves and left ulnar nerve) and minor denervation in the muscles supplied by the affected nerves, consistent with MM.

Dermatology team diagnosed the skin lesions as nodular prurigo.

### Treatment, outcome and follow up

As pain and weakness significantly worsened during the following two weeks and a provisional muscle and nerve biopsy description suggested an inflammatory process, a course of intravenous (iv) cyclophosphamide (15 mg/kg) and oral prednisolone (1 mg/kg/d) were started with marked strength improvement in the following days. Oral cyclophosphamide (150 mg/day) was started two weeks later.

Final biopsy findings described a granulomatous vasculitic process associated with axonal neuropathy. Muscle biopsy showed perivascular inflammation without fibrinoid necrosis and a cluster of cells resembling a loose non-necrotic granuloma. Nerve biopsy revealed florid axonal neuropathy with large and small myelinated fibers active degeneration, dense inflammatory infiltrates including several scattered eosinophils and aggregates of epithelioid macrophages forming loose granulomata in perineurium and endoneurium, without vessel fibrinoid necrosis. Skin biopsy was unremarkable.

Granulomatosis with polyangiitis (GPA) was presumed as histology identified eosinophils and poorly formed granulomata. She was discharged on cyclophosphamide, analgesia and prednisolone taper until 20 mg/day. Clinically she was almost pain free, but suffered severe fatigue.

Within three months, she stopped medication against medical recommendation and worsened in the following eight weeks, with severe neuropathic pain and inability to walk autonomously. Neurological examination revealed grade 3/5 tetraparesis, lower limbs arreflexia and sensory gait ataxia. Inflammatory markers were increased (ESR = 86 mm/h; CRP = 54 mg/L). Cyclophosphamide (150 mg/day) and prednisolone (40 mg/day) were restarted with clinical and laboratorial improvement within one month. Nevertheless, recurrence of severe symptoms occurred two months later and alemtuzumab (20 mg/day, iv, 5 days) was used with only mild improvement within three weeks.

By this time, 150 mg/day azatioprine (AZA) was associated with prednisolone taper, but worsening occurred on every taper attempt. Whole body PET performed during an exacerbation with severe systemic symptoms excluded other areas of inflammatory activity and neoplasms.

After four months of AZA, slow clinical recovery was reported. Stability was achieved for further six months on prednisolone (17,5 mg/20 mg on alternate days) and AZA (175 mg/day). However, severe pain and weakness worsening followed and AZA was switched to mycophenolate mofetil (MMF) (750 mg bd). At that point, breast implants were removed as bilateral breast tumefaction and a large left axillary adenopathy were noticed. Biopsy showed granulomatous lymphadenitis and patchy stromal fibrosis of breast tissue with periaqueductal and perilobular inflammation including occasional poorly formed non-necrotizing granulomas, without signs of malignancy and with negative acid-fast bacilli and fungi stains.

After four months of stability, neurological symptoms recurrence associated with bilateral uveitis were treated with iv methylprednisolone (1 g, 3 days). Oral prednisolone (40/60 mg on alternate days) and MMF (1 g, bd) were increased. Four rituximab infusions (375 mg/m^2^/week) were performed. Despite improvement of systemic symptoms and inflammatory markers, marked disability persisted due to pain and weakness.

Skin biopsy obtained three months later, during a period of skin lesions exacerbation, showed numerous granulomata in the dermis with perivascular lymphocytic infiltrates, without vasculitic features. This time the clinical and histopathological features suggested sarcoidosis, so our treatment strategy changed.

**Figure 1:**
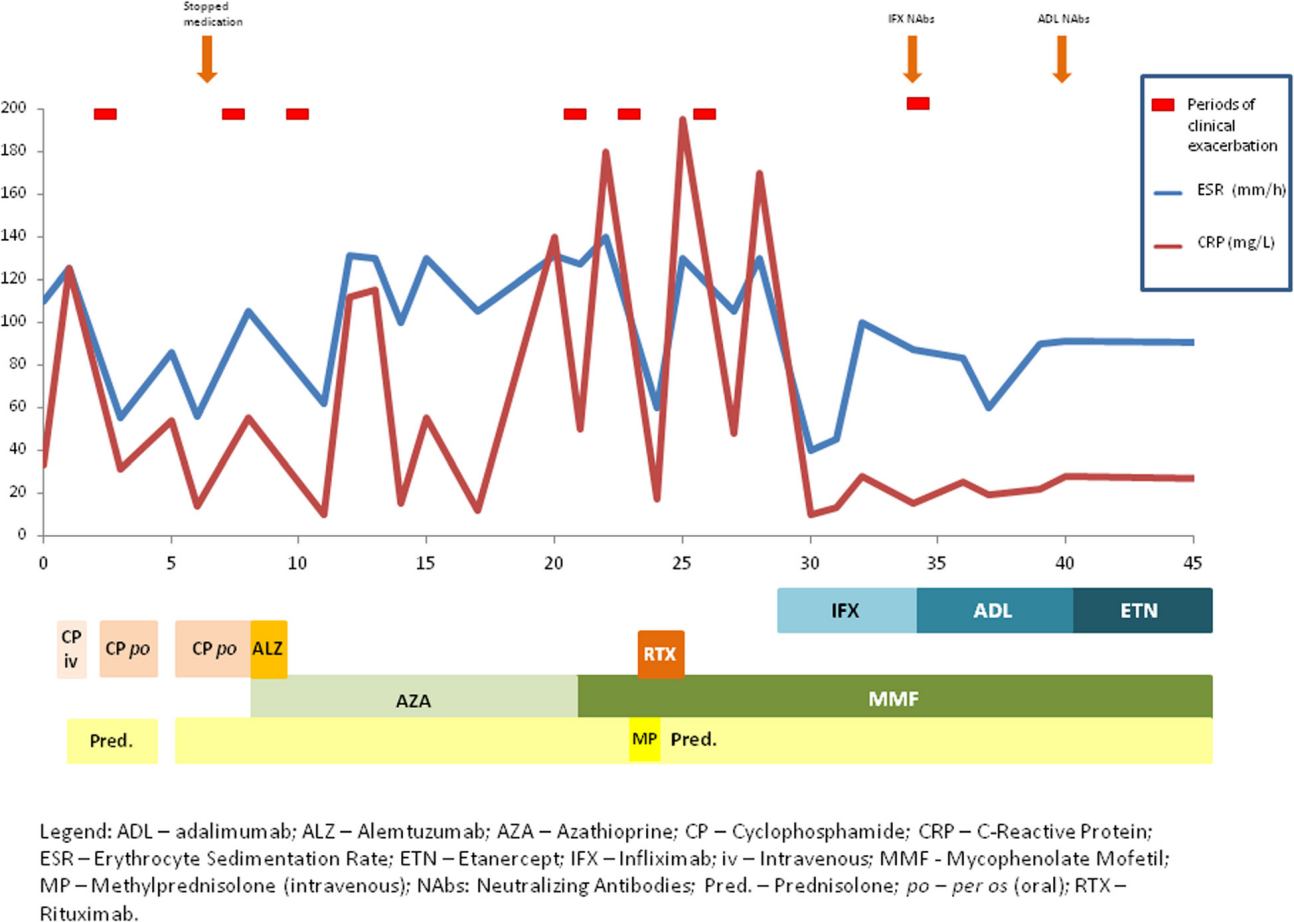
**Clinical and inflammatory markers evolution during the course of the disease and treatments performed during this period.**

Infliximab (3 mg/kg, iv, at 0/2/6 weeks and every 6 weeks thereafter) was started in combination with MMF and prednisolone with excellent prompt clinical and laboratorial response. Coinciding with clinical symptoms recurrence, five months later, NAbs againts infliximab were detected. Switch to adalimumab (40 mg, sc, every second week) maintained improvement, however, despite strength improvement, sensory ataxia and neuropathic pain still hindered autonomous walking. Eight months later, another period of treatment efficacy loss followed, associated with NAbs against adalimumab, reducing the choice of TNF-α inhibitors described as beneficial in sarcoidosis. We eventually chose etanercept (50 mg, sc, once a week) still in combination with MMF (1.5 g/day) and prednisolone (40 mg/60 mg on alternate days) which controlled most clinical symptoms with complete motor and cognitive functional recovery. She remains clinically stable after eighteen months on etanercept, presenting full power with fatigability, fluctuant facial asymmetry, marked sensory gait ataxia, areas of sensory deficits with numbness and dysesthesia, absent reflexes in the lower limbs and decreased reflexes in the upper limbs. Despite clinical stability, inflammatory markers remain increased (ESR = 83 mm/h; CRP = 14 mg/L) and skin lesions persist.

Figure 1 represents clinical symptoms and inflammatory markers evolution during the disease course and the treatments performed during this period.

## Discussion

Sarcoid neuropathy is a rare manifestation of sarcoidosis, estimated to occur in 15% of cases [14]. Theories to explain sarcoid neuropathy include ischemic axonal degeneration and demyelination resulting from local pressure provoked by epineural and perineural granulomas and granulomatous vasculitis; axonal and myelin damage by proteolytic enzymes secreted by epithelioid cells; and ischemic nerve lesions resultant vessel fibrinoid necrosis [3]. Diagnosis requires histological demonstration of non-caseating granulomas, preferably in biopsy tissue obtained from other organs involved, reserving nerve biopsy for patients without other accessible organs affected. As no histological features differentiate sarcoid granulomas, other granulomatous diseases must be excluded, most importantly acid-fast bacilli and fungi infections. In the case of our patient, MM associated with ANCA antibodies and granulomatous vasculitic process suggested a systemic vasculitis, possibly GPA or Churg-Strauss syndrome (CSS). Despite disparate manifestations, these conditions can present with MM before evident systemic features emerge [15,16]. Histologically, GPA usually shows vasculitis and/or necrotizing granulomas and CSS characteristically reveals eosinophilic infiltrates, vasculitis and granulomas with eosinophilic necrosis [17]. GPA usually associates with c-ANCA PR3 specific and CSS with p-ANCA MPO specific [17]. Atypical ANCA occur in various disorders, including systemic immune-mediated diseases and infections [17]. In our patient, atypical ANCA without PR3/MPO specificity and absence of necrotizing granulomas were uncharacteristic for systemic granulomatous vasculitis.

Infectious granulomatous diseases that may present with MM were also considered in the differential diagnosis. Tuberculoid Leprosy (TL), caused by *Mycobacterium leprae,* usually involves the skin and the peripheral nerves in an asymmetrical pattern and may present as MM [18-20], however the skin lesions are usually anesthetic and present as hypopigmented macules or erythematous plaques with well-defined elevated borders and an atrophic center, and peripheral nerves are usually tender and thickened, making this diagnosis less likely in our patient. TB can also very rarely present with peripheral neuropathy, but usually results from nerve or root compression by regional tubercular lymphadenitis, vertebral collapse or abscess, without direct invasion of the peripheral nerves by the necrotizing granulomas [21-23]. The nerve biopsy of our patient showed dense inflammatory infiltrate in all nerve compartments with granulomata in the perineurium and endoneurium, findings not expected in TB. The negative stains for a*cid-fast bacilli* and mycobacterial cultures were also not suggestive of these granulomatous infections.

The recently described Autoimmune/Inflammatory Syndrome Induced by Adjuvants (ASIA) [24], includes immune mediated diseases triggered by an adjuvant stimulus. It results from exposure to external stimuli, including infectious agents, vaccines, silicone, aluminium salts and others, that are thought to stimulate the immune system and trigger an immune response in persons with a favourable genetic background. The most frequently reported symptoms are myalgia, arthralgia, fatigue, sleep disturbances, fever, cognitive impairment and other neurological manifestations, especially associated with demyelination, and this symptoms are thought to improve with removal of the inciting agent [24,25]. As our patient had breast implants, ASIA was considered in the differential diagnosis, however the clinical manifestations were not the most typical of this syndrome and the disease exacerbations continued after surgical removal of the breast implants.

Sarcoid neuropathy early treatment with oral prednisolone (1 mg/kg/day) or iv methylprednisolone (0.5-1 g/day) is recommended and steroid-sparing medications, such as AZA or MMF, must be considered. In refractory patients, TNF-α inhibitors, mostly infliximab are proposed [26], as TNF-α is thought to be involved in antigen-driven, cell-mediated responses and in granuloma formation. Monoclonal antibodies, infliximab and adalimumab, bind both soluble and membrane bound TNF-α, whereas etanercept binds only soluble TNF-α [13]. Reports of TNF-α inhibitors induced sarcoidosis [27], paradoxically describe etanercept as being more commonly associated with granuloma development [28], possibly because of incomplete TNF-α blockage. NAbs usually reduce anti-TNF-α antibodies efficacy [29], and immunossupressants are suggested to reduce antibody development risk, however our patient developed NAbs despite concomitant MMF. Antibodies to etanercept are usually non-neutralizing and do not affect efficacy or safety [30].

In the case of our patient, steroids, cyclophosphamide, alemtuzumab, azathioprine, mycophenolate mofetil and rituximab were used, all with transient and insufficient response. Alemtuzumab was used for presumed granulomatosis with polyangiitis, before sarcoidosis diagnosis, as it has shown benefit in ANCA-associated refractory vasculitis [31] and clinical trials are ongoing to show evidence. Despite good clinical and laboratorial response was achieved with infliximab and, later, adalimumab, neutralizing antibodies developed to both drugs, so etanercept was tried with good clinical response.

To the best of our knowledge, this is the first report of sarcoidosis responding to etanercept. In our patient, despite significant clinical improvement, inflammatory markers, especially ESR, remain increased. The lack of alternatives with proven efficacy and the reasonable clinical stability achieved support our decision to maintain this treatment.

## Conclusions

Mononeuritis multiplex is a rare presentation of sarcoidosis with rare cases described in the literature. The differential diagnosis is extensive and biopsy of the nerve, or other affected organ, is crucial for diagnosis. In refractory patients, TNF-α inhibitors are invaluable, with many successful cases of sarcoidosis treatment with infliximab and adalimumab reported in the literature. This article presents the first report of sarcoidosis treated with etanercept, adding this drug as an option for refractory patients who failed other TNF-α inhibitors, having the advantage of not being associated with development of neutralizing antibodies.

### Learning points/take home messages

- Mononeuritis multiplex may be a presentation of sarcoidosis,
- Sarcoidosis diagnosis requires histological demonstration of non-caseating granulomas, preferably in biopsy tissue obtained from organs involved outside of the nervous system,
- TNF-α inhibitors, mostly infliximab and adalimumab, are reported as efficacious in refractory cases,
- Etanercept may be an option in patients who failed other TNF-α inhibitors, having the advantage of not being associated with development of neutralizing antibodies.

## Patient consent

Written informed consent was obtained from the patient for publication of this Case report and any accompanying images. A copy of the written consent is available for review by the Editor of this journal.
